# Supplementary material for: Cost-Effective Detection of SNPs and Structural Variations in Full-Length Genes of Wheat and Sunflower Using Multiplex PCR and Rapid Nanopore Kit
Source: Biology (Basel). 2025 Jan 29;14(2):138. doi: 10.3390/biology14020138 (PMC11851361; doi:10.3390/biology14020138)
Supplement: Supplementary file 1 [file biology-14-00138-s001.zip › Supplementary figures for amplicons_2024_1.pdf]

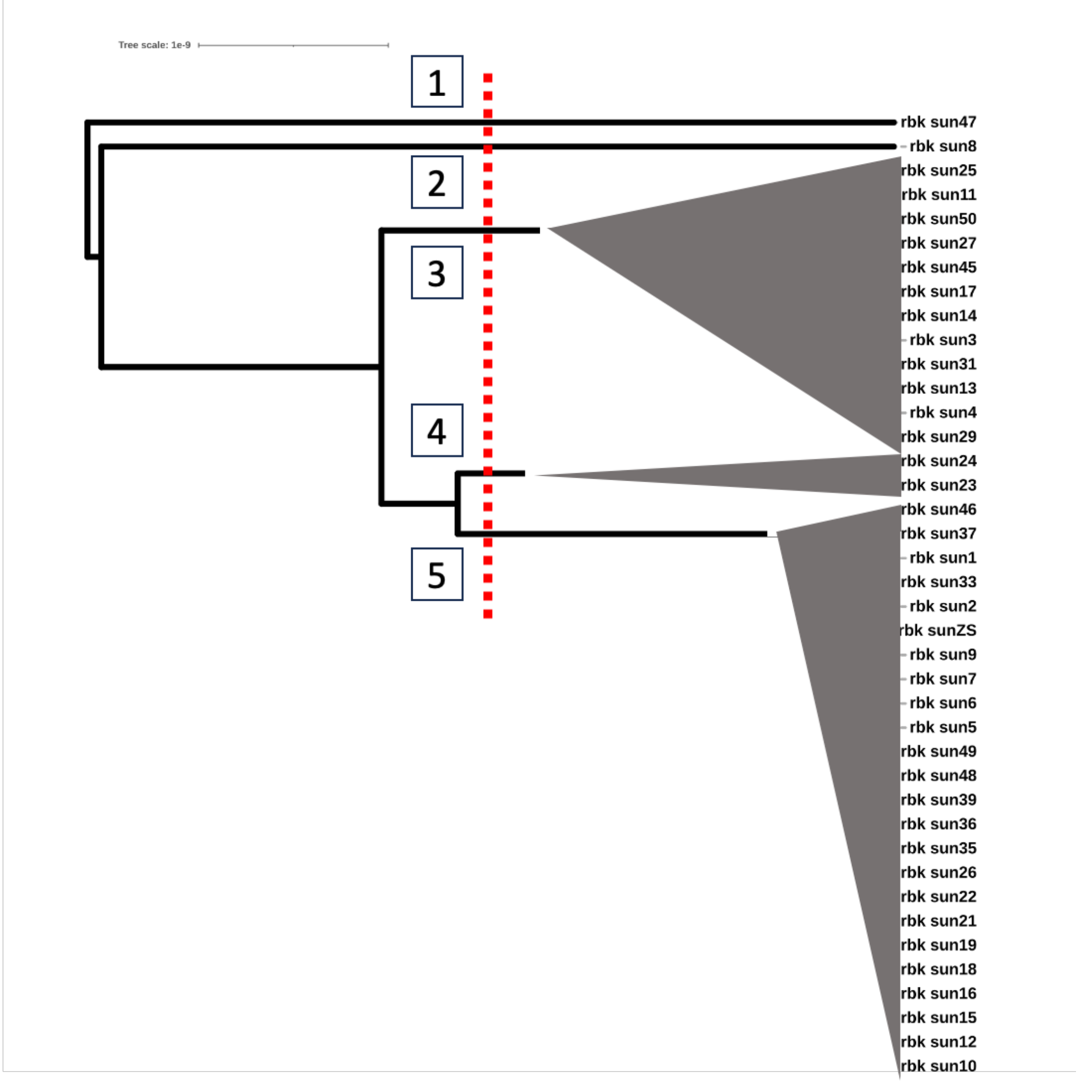


**Supplementary Figure S1.** Phylogenetic tree of Ahasl1 genes of sunflower genotypes build using SNPs data for. Five divergent groups are indicated by numbers.

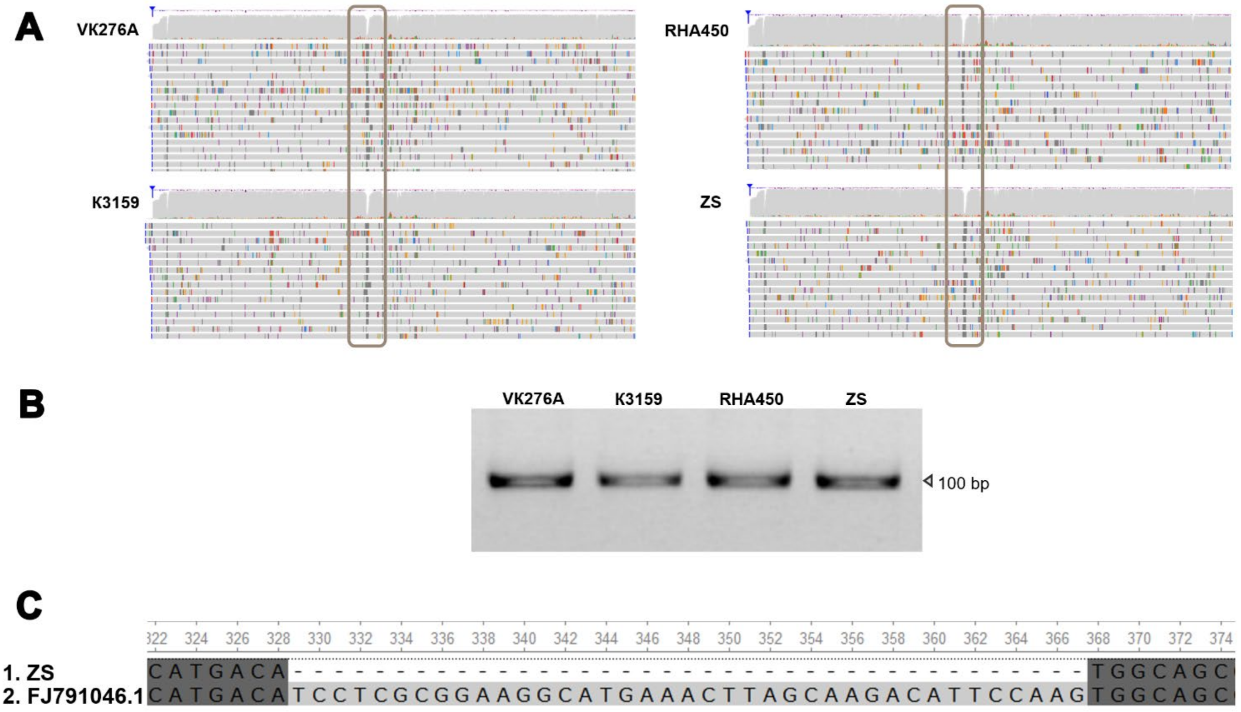


**Supplementary Figure S2.** Sequence analysis of FAD2 gene helps to match the reference sequence with the obtained reads for sunflower for deletion identifying (A). (B) PCR results with specific primer pairs flanking the deletion for sunflower samples. (C) Identification of deletion after multiple alignment of sequences obtained by Sanger sequencing.
